# Developmental or degenerative – *NR2E3* gene mutations in two patients with enhanced S cone syndrome

**Published:** 2011-02-17

**Authors:** Nitin Udar, Kent Small, Meenal Chalukya, Rosamaria Silva-Garcia, Michael Marmor

**Affiliations:** 1 Department of Ophthalmology, University of California Irvine, Irvine, CA; 2 Molecular Insight LLC, Cedars-Sinai Medical Center, Los Angeles, CA; 3 Department of Hematology and Oncology, University of California Los Angeles, Los Angeles, CA; 4 Department of Ophthalmology, Stanford University Medical Center, Stanford, CA

## Abstract

**Purpose:**

Enhanced S Cone Syndrome is a rare autosomal recessive disorder characterized clinically by an absence of rod function, a replacement of most L and M cone function by S cone activity (Goldmann-Favre Syndrome) and by variable degrees of retinal degeneration in different families. The causative gene, nuclear receptor subfamily 2, group E, member 3 (*NR2E3*), controls the developmental sequence for rods and cones. The purpose of this study was to compare the nature and implications of mutations in two subjects with Enhanced S Cone Syndrome who have significantly different degrees of degenerative damage.

**Methods:**

A direct sequencing approach was used to identify the mutations. Genomic DNA was amplified from all the exons of *NR2E3* and used as a template for sequencing. Of the two families studied, Case 1 is of Persian ethnicity while Case 2 is Brazilian. A total of six individuals within the two families were studied.

**Results:**

Case 1 (original propositus of the syndrome) has the characteristic developmental rod/cone abnormality with large amplitude electroretinogram responses and no retinal degeneration. She was homozygous for a novel mutation, c.[del196–201del6] (p.G66-C67del), which lies entirely within the P-box for this gene. By comparison, Case 2 had Goldmann-Favre Syndrome with retinal degeneration and low electroretinogram signals. She was a compound heterozygote for c.[119–2A>C]+[del194–202del9] (p.N65-C67del), mutations that have been reported previously. Her second mutation overlaps that of Case 1 within the P-box.

**Conclusions:**

The novel in-frame homozygous deletion of Case 1, within the P-box motif of the DNA binding domain, caused a developmental abnormality without retinal degeneration. Case 2, with more traditional Goldmann-Favre Syndrome with retinal degeneration, was a compound heterozygote where one allele had a similar P-box deletion but the other was a splicing defect. Case 1 is the first reported homozygous deletion within the P-box. This is the first report of *NR2E3* mutations in a Persian and a Brazilian family.

## Introduction

Enhanced S Cone Syndrome (ESCS) is a rare autosomal recessive retinal disorder that overlaps clinically with the more severe Goldmann-Favre Syndrome. It is characterized clinically by an absence of rod function and a replacement of most L and M cone activity by S cone responsiveness [1-3]. The diagnosis is determined by characteristic electroretinogram (ERG) findings of no rod activity and large S cone–mediated responses under both photopic and scotopic conditions. There may also be retinal degenerative changes in a circular pattern about the arcades, and cystic edema in the fovea, which carry the designation of Goldmann-Favre Syndrome and are associated with a considerable loss of visual acuity and visual field [2,3]. There is debate as to whether ESCS is largely static or progressive, and there is variability among different families [4-8].

The gene for ESCS on chromosome 15q23 was originally identified by Haider et al. [9], and was termed nuclear receptor subfamily 2, group E, member 3 (*NR2E3*). It encodes a nuclear receptor ligand-dependent transcription factor. The expression of this gene was localized to the outer nuclear layer of the human retina [10,11], as well as other animal models [12-14]. This influences the development of photoreceptors and their differentiation into rod and cone types [15-18]. In ESCS, rods fail to develop and are replaced largely by cells that behave physiologically as S cones. L and M cones are reduced in number. Different abnormalities in the *NR2E3* gene have been reported, and individuals with ESCS manifestations may be either homozygous or heterozygous. Most of these reports involve patients with significant levels of retinal degeneration and visual dysfunction. *NR2E3* mutations including the common mutation c.119–2A>C have been reported in clumped pigmentary retinal dystrophy [19]. Autosomal dominant/recessive retinitis pigmentosa patients also have been shown to carry the common p.G56R (RP37) mutation [20-22].

We had access to the original propositus [1], a unique case that might elucidate the distinction between photoreceptor developmental abnormality and retinal degeneration. This case was first described because of her unusual ERG, showing large amplitude signals with a characteristic S cone pattern but with essentially no degenerative changes. We have sequenced the gene from her family as well as that from another family with more typical Goldmann-Favre Syndrome, to see if the results might give clues to the phenotypic variation in ESCS (development versus degenerative change). It raises the intriguing question of how certain in frame deletion mutations within the ligand binding domain results in a developmental only defect.

## Methods

The study was approved by the Institutional Review Board of the University of California Los Angeles (UCLA-HSPC 94–07–243–03). Informed consent was obtained from participants and the study was performed according to the tenets of the Helsinki Declaration. The patients were given full ophthalmological exams, and ERGs were performed according to the International Society for Clinical Electrophysiology of Vision (ISCEV) standards.

### Samples

A total of six individuals from two families (Family 1: #2743, 3385, 3386, 3387 and Family 2: #2883, 2884) in good health were studied. DNA was extracted from whole blood lymphocytes, using the Gentra blood extraction kit (Qiagen, Chatsworth, CA). PCR amplification of the genomic DNA was performed using intronic primers to the human *NR2E3* gene (Table 1). The PCR products were separated on agarose gel; bands were cut and then purified using a Qiagen DNA gel purification kit (Qiagen).

**Table 1:** Primer sequence

**Primer**	**Primer sequence (5′-3′)**
NR2E3–1AF	AGCATGGGGTAGCAGGACTGAC
NR2E3–1AR	TTGGTCTGGTCTCCATGGGTTAC
NR2E3–1BF	GGCAGCTCCTGAGTTCAGACAGA
NR2E3–1BR	CTGAGTTGTTCTGGCTCCTTCCA
NR2E3–2F	GTTCGTTCAAATGCGGGTGAG
NR2E3–2R	GGTCAGTGTCCCTCCCATGC
NR2E3–3F	AGGGGTTCTGGAGGGGTGAG
NR2E3–3R	GGACTCAGTGTTGGACTCCATGC
NR2E3–4AF	CAGGCGGGGATGAACCAG
NR2E3–4AR	TTATAAGGCTGGCCATGAAGTGG
NR2E3–4BF	GCATGGAGTCCAACACTGAGTCC
NR2E3–4BR	TGTGATCTTAGCGCCTGCTTCTC
NR2E3–5AF	AGGATGGTGAGTGGGAGAGCAG
NR2E3–5AR	ATGAAGAGTAGGCGAGCCGAGGT
NR2E3–5BF	CCTGAGTTCCCCTCCTCTCCATA
NR2E3–5BR	ATCACCATCCAAGCTGTGTGCAT
NR2E3–6AF	CTGGCTGATGTCAGGAGAGCATT
NR2E3–6AR	CCGGAACCGAGAGATAGTTTCCT
NR2E3–6BF	GGCGTGGAGTGAACTCTTTCTCC
NR2E3–6BR	AGTCCAGCCTCACCACTCTCCTC
NR2E3–7AF	CAGAGCCCACCCCACAGG
NR2E3–7AR	TGAACTGAGACCCTTGTGCTGTC
NR2E3–7BF	CCCGTGAGGTGACCTGAGCAT
NR2E3–7BR	CAAAGTCCCTCCCAATTCTGCTT
NR2E3–7CF	CCCTAGCCAGGTACTGAGGGTTG
NR2E3–7CR	AGCCCTGTGTATGACCCTCTGCT
NR2E3–7DF	AGCCCGTTCAGGACTTTGAATG
NR2E3–7DR	TCCATGTGCTTGGCATCTCTACA
NR2E3–8AF	ATGTGGCTTTTCCTCGAAATTCCT
NR2E3–8AR	CCATCAATATACAGTTTGGGGCTAT
NR2E3–8BF	GCAATTCCTCGTAGGTGTGTGTACC
NR2E3–8BR	TGCCCAGATCAAAATCAACATTTCT
NR2E3–8CF	TATGCAGAGTTCAGGAACAGGCAAG
NR2E3–8CR	GGGTGGTTGAATTCTATGGGAGATT

### DNA sequencing

The purified DNA was sequenced using a Thermo Sequenase Radiolabeled Terminator Cycle Sequencing Kit (USB, Cleveland, OH) and electrophoresed on a 6.5% acrylamide gel containing 7 M urea and 1× TBE containing 90mM Tris, 45 mM Tris-borate and 1 mM EDTA pH 9.5 on a DNA sequencing apparatus (Biorad, Hercules, CA) at 80 W in 1× TBE.

The *NR2E3* reference sequence NM_014249.2 was used as the reference RNA sequence and the AJ276674 genomic assembly was used for the DNA sequence.

## Results

### Case 1 (propositus)

#### Clinical findings

**Figure 1:**
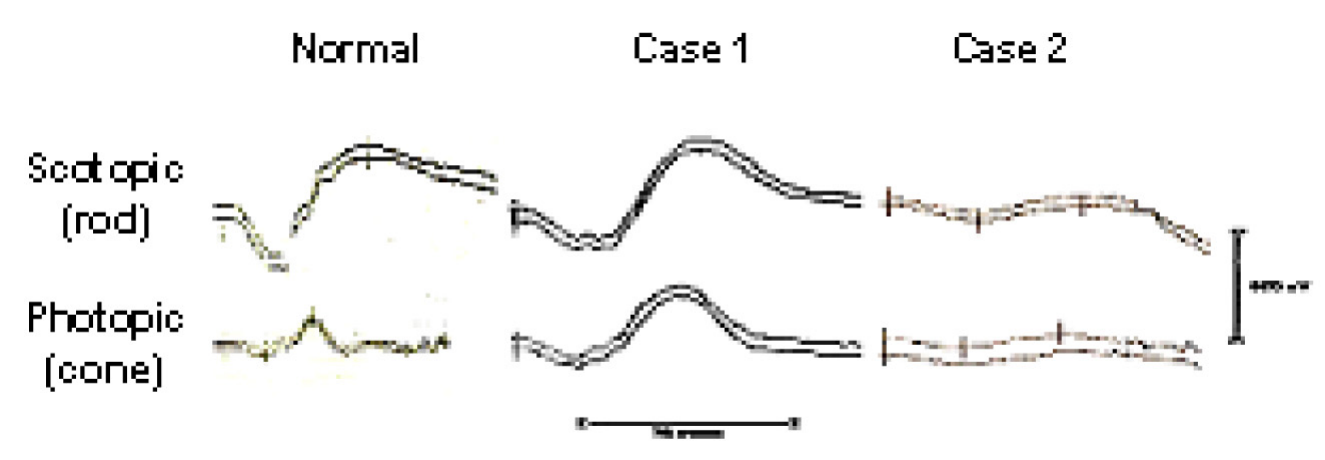
Electroretinograms comparing normal tracings to Case 1 and Case 2. Case 1 shows electroretinogram (ERG) amplitudes similar to the normal dark-adapted combined rod-cone response under both scotopic and photopic conditions (Scale bars show amplitude 500 microvolts, duration 75 ms). The responses from Case 2 have similar waveform, and scotopic-photopic homology, but lower amplitude.

The propositus of ESCS is a Persian female (#2743) first seen at age 10 years because of night blindness. No other immediate family members were reported to be affected. She was otherwise asymptomatic, and had a visual acuity 20/25 on the right, 20/20 on the left. Visual fields and color vision were normal, and a retinal exam was normal except for a few sparse yellowish spots in the arcade regions. Vascular caliber was full, and she had a normal foveal reflex. Her ERG showed no detectable rod signal, but large amplitude slow scotopic responses that hardly changed under photopic conditions were observed (Figure 1) [1]. These findings were stable on follow-up nine years later [4]. Multifocal ERG testing showed L and M cone function mixed in with the S cone responses in the central 9° of the macula. The individual represents an unusual extreme of ESCS in which there are essentially no stigmata of the retinal degeneration of the Goldmann-Favre Syndrome.

#### Genetic findings

**Figure 2:**
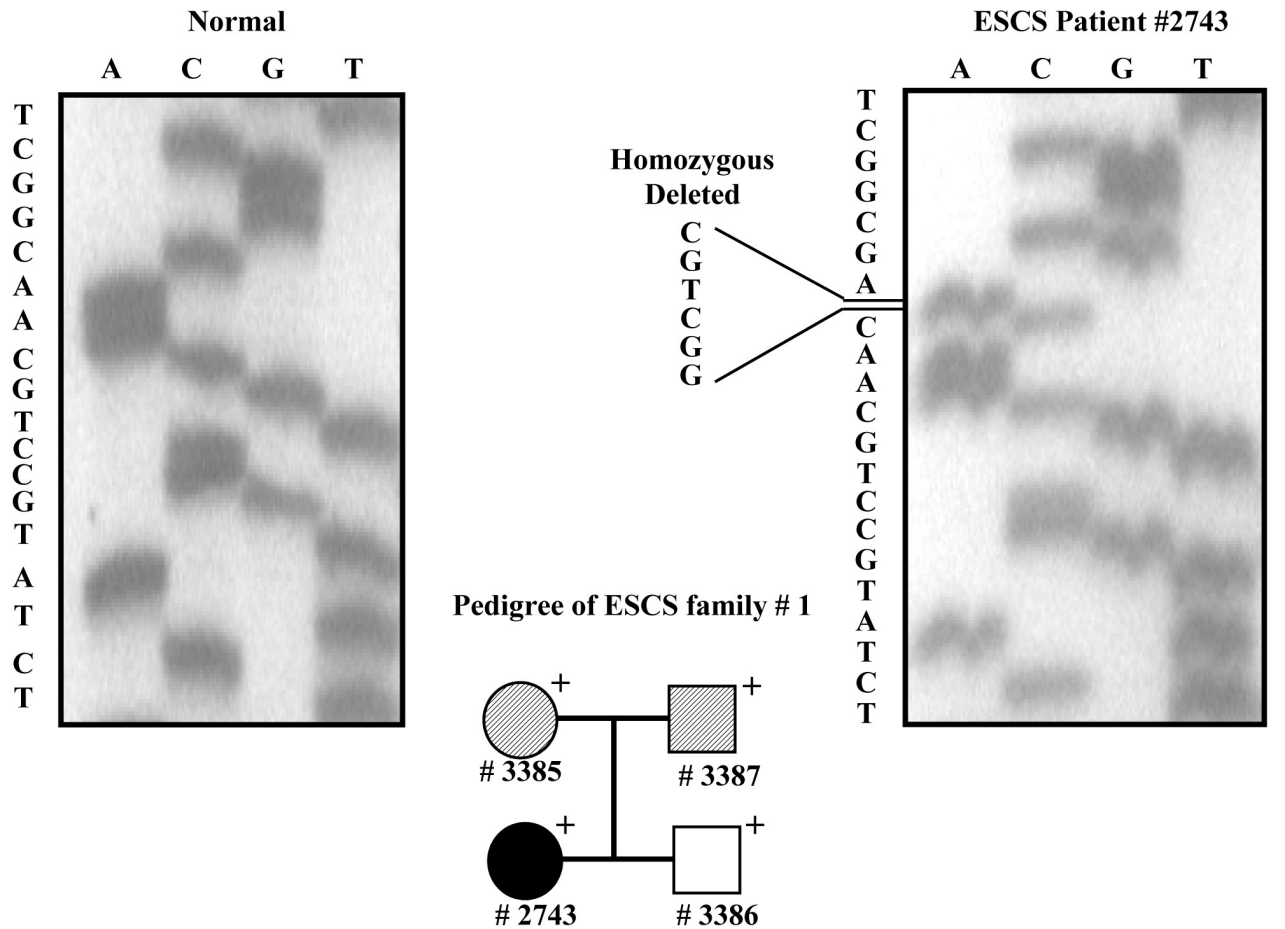
DNA sequencing of the *NR2E3* gene in family 1 with the propositus #2743 showing the homozygous deletion. Pedigree: + - indicate individual that have been examined. Black solid fill ● – affected phenotype. White solid fill □ – normal phenotype. Pattern filled square and circle– individual assumed carrier. # - Sample Number.

**Figure 3:**
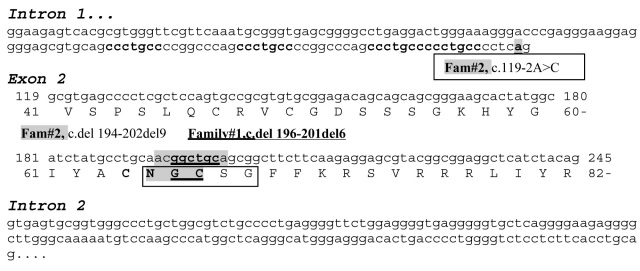
Sequence of intron 1 and exon 2 of *NR2E3* showing the mutations of Case 1 and 2. Repeat sequences are given in bold. The novel mutations of Case 1 are underlined. The mutations of Case 2 are shaded. A square box defines the P-box.

After amplification of all exons of *NR2E3*, we sequenced them for patient #2743. Using these data, we identified a homozygous in-frame deletion mutation c.del196–201del6 (p.G66-C67del; Figure 2). We amplified exon 2 for all three family members (parents #3385, #3387 and brother #3386; Figure 2). None of the family members had the ESCS phenotype. DNA sequencing results revealed that both the parents (#3385 and #3387) were heterozygous for the mutation (data not shown). The patient’s brother (#3386) was negative for the mutation (data not shown), suggesting that he inherited the normal allele from both parents. As shown in Figure 3, this novel mutation is located within the conserved P-box of the DNA binding domain.

### Case 2

#### Clinical findings

This 29-year-old Brazilian woman had been night blind all of her life, and had poor vision in the right eye for many years [23]. Her parents were not affected. Visual acuity was 20/400 on the right and 20/40 on the left. Fundus examination showed mildly attenuated arteries and a zone in the midperiphery of diffuse depigmentation and faint yellow spots. The central macula was grossly cystic on the right but minimally so on the left. She had a chronic fluctuating uveitis and vitritis in the right eye, which affected her visual acuity, but never had uveitic attacks on the left. Goldmann visual fields were not testable on the right because of poor vision, and showed generalized constriction on the left. Her ERG responses were similar in the two eyes, and showed no detectable rod responses. However, she had slow and low amplitude b-waves to a strong flash, of similar waveform, under both scotopic and photopic conditions (Figure 1). The ERG was unchanged on a follow-up examination two years later. Her clinical diagnosis was Goldmann-Favre Syndrome.

#### Genetic findings

**Figure 4:**
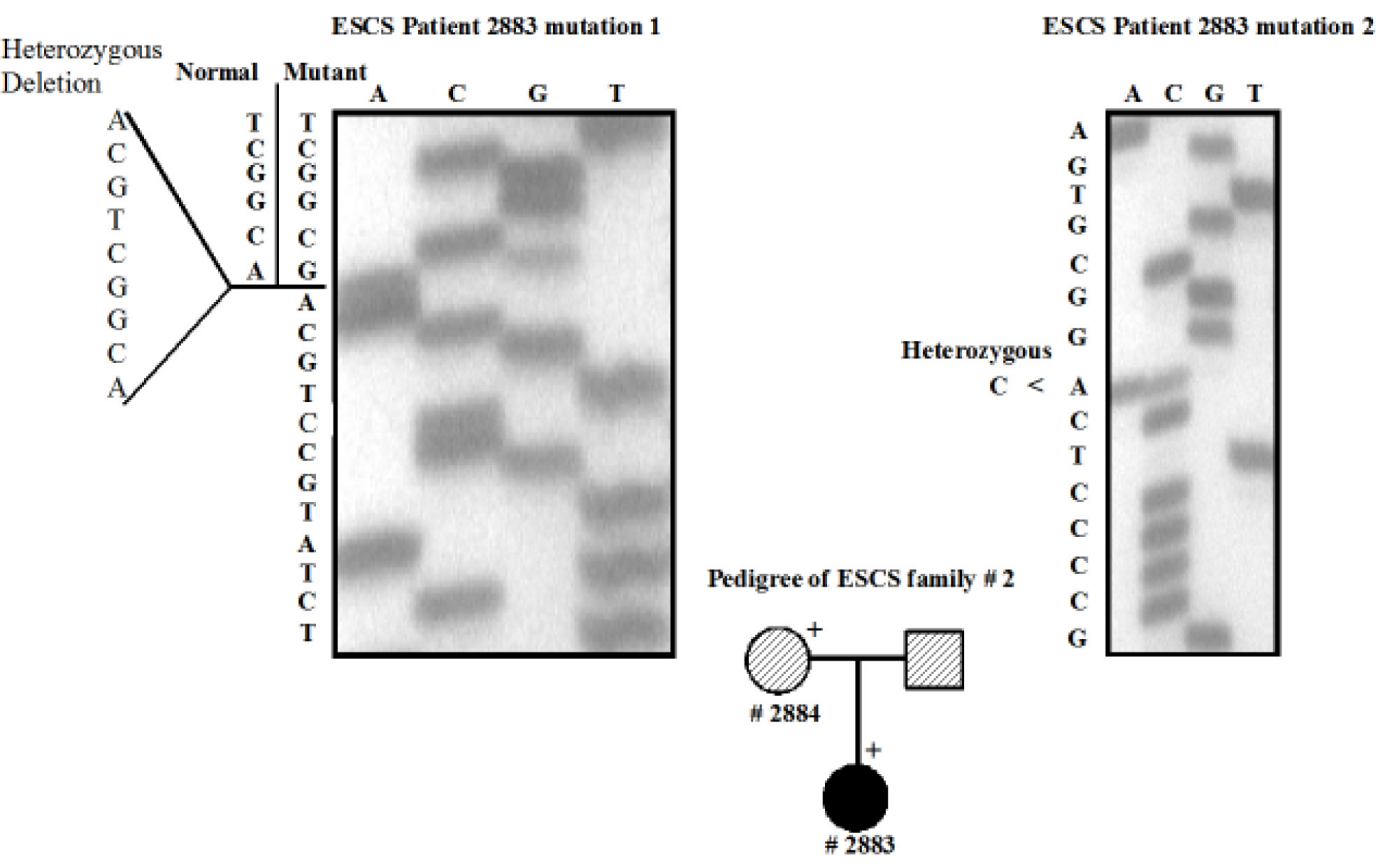
DNA sequencing of the *NR2E3* gene in family 2. Individual #2883 illustrates the compound heterozygous mutation. Pedigree: + - indicate individual that have been examined. Black solid fill ● – affected phenotype. White solid fill □ – normal phenotype. Pattern filled square and circle– individual assumed carrier. # - Sample Number.

Sequencing of all exons was performed after amplification of genomic DNA for the patient #2883 (Figure 4). The results revealed that the patient has a compound heterozygous mutation, which was c.[119–2A>C]+[del194–202del9] (Figure 4). The mother (#2884) was heterozygous for the mutation c.del 194–202del9 (data not shown) and a normal allele. As shown in Figure 3, this mutation is located within the conserved P-box of the DNA binding domain. We were unable to obtain DNA from the father. Patient #2883 could have inherited the c.[119–2A>C] mutation from the father, or it could have been a de novo event. This combination of mutation has previously been reported in another case [9,19].

## Discussion

From a clinical standpoint, our two cases are quite different. Case 1, which has been described previously in electrophysiological studies [1-3], lies at one extreme of the ESCS complex, being essentially free of Goldmann-Favre stigmata and without evidence of degeneration. The patient’s ERG signals are of comparable amplitude to a normal “mixed rod-cone” ERG, which suggests the presence of functional S cones in numbers comparable to the photoreceptor density of the normal retina. This striking developmental abnormality results from a small deletion of “ggctgc” at position c.196–201 (see Figure 3), which lies within the P-box region that has been described for this gene [12-14,24]. The P-box of nuclear receptors is responsible for the interactions of nuclear receptors with specific response elements in DNA upstream of target genes, and thus the control of gene expression. It is perhaps relevant that there is a similar “gcctgc” sequence at position c.187, and a “ggcttc” at position c.205. The sequence can also be viewed as a direct repeat TGCA(A/G)CGGC at position 190 and 199 within the P Box.

The spectrum of clinical phenotypes with *NR2E3* abnormalities is wide, and includes clumped pigmentary degeneration and autosomal dominant retinitis pigmentosa as well as ESCS and Goldmann-Favre Syndrome [20,22,25,26]. Three recent reports have discussed possible mechanisms by which the degenerative component of the ESCS complex may vary greatly [7,27,28]. Cases with surprisingly little degenerative change have been reported with variations of the c.119–2A>C mutation [27,28]. Our report adds to this growing body of evidence that mutations in this gene lead to a developmental defect and/or degenerative changes. Whereas the P box deletion [del194–202del9] of Case 2 has previously been reported with varying phenotypes [7,9,19,21,27], we believe Case 1 is the first homozygous deletion within the P-box to be reported, and to define this locus as possibly developmental (although we recognize that a single case cannot confirm this conclusion).

Case 2 would be diagnosed as Goldmann-Favre Syndrome, although not a terribly severe example. The individual is somewhat similar to the “mild phenotype” reported by Hayashi et al. [29] in a Japanese patient with two missense mutations. Her chronic uveitis in one eye has not been reported as a component of this syndrome, and could be a new or an unrelated finding. It is interesting that her foveal cystic changes were markedly accentuated as a result of the uveitis, and visual acuity was only mildly reduced in the left eye where cystic changes were minimal. This individual exhibited the splice junction c.[119–2A>C], which is responsible for her developmental as well as degenerative changes. The c.119–2A>C change is the most common mutation found in ESCS, autosomal recessive retinitis pigmentosa (arRP), and clumped pigmentary retinal dystrophy patients [7,9,19,30].

The second mutation in Case 2 is also located within the P-box. This is the first ESCS mutation reported in a Brazilian patient. Due to nomenclature recommendations, this mutation is designated as c.[119–2A>C]+[del194–202del9 (p.N65-C67del)]. However this does not rule out the possibility that this mutation is actually [119–2A>C]+[del 190–198del9] (p.C64-G66del). Results using Sanger DNA sequencing in either direction would essentially give identical results for either of the two above mentioned mutations due to the presence of a short sequence repeat. However the amino acid translations are different for the above-mentioned mutations. Therefore, one has to be cautious in assuming the nature of p.N65-C67del versus p.C64-G66del mutation in all reported cases. Although functional assays have been developed by Kanda et al. [31], these may not be able to differentiate between these changes at the functional level.

We hypothesize that the NR2E3 protein could have functions beyond the developmental process that are independently controlled and lead to degeneration. We also hypothesize that the presence of direct repeats near the 5′ end of exon 2 and another repeat within the P-box may lead to looping structures in the DNA within these two respective regions. A consequence of this could lead to errors in replication [20]. These are de novo events and are not related to mutations that are inherited.

This report brings the total number of mutations overlapping this region at amino acid position p.N65-C67 to seven reports in seven different families [9,19,21,27]. The synonymous variation at position c.195 C>T, N65N reported by Bernal et al. [30] overlaps with this region as well. It is possible that these mutations are random events and not specific for a family/ethnicity; however, this will need to be verified in the future by genotyping these samples with closely linked markers. We hope that further studies on the phenotypic variability of this syndrome will enhance the understanding of how both retinal development and retinal degeneration are controlled.
